# Lower Versus Higher Oxygenation Targets for Critically Ill Patients: A Systematic Review

**DOI:** 10.7759/cureus.41330

**Published:** 2023-07-03

**Authors:** Ahmed M Abdelbaky, Wael G Elmasry, Ahmed H. Awad

**Affiliations:** 1 Intensive Care Unit, Dubai Academic Health Corporation - Rashid Hospital, Dubai, ARE

**Keywords:** stroke, oxygenation, myocardial infarction, intensive care unit, hyperoxia, critically ill

## Abstract

Supplemental oxygen is a standard therapeutic intervention for critically ill patients such as patients suffering from cardiac arrest, myocardial ischemia, traumatic brain injury, and stroke. However, the optimal oxygenation targets remain elusive owing to the paucity and inconsistencies in the relevant literature. A comprehensive analysis of the available scientific evidence was performed to establish the relative efficacy of the lower and higher oxygenation targets. A systematic literature search was conducted in PubMed, MEDLINE, and Scopus databases from 2010 to 2023. Further, Google Scholar was also searched. Studies evaluating the efficacy of oxygenation targets and the associated clinical outcomes were included. Studies that included participants with hyperbaric oxygen therapy, chronic respiratory diseases, or extracorporeal life support were excluded. The literature search was performed by two blinded reviewers. A total of 19 studies were included in this systemic review, including 72,176 participants. A total of 14 randomized control trials were included. A total of 12 studies investigated the efficacy of lower and higher oxygenation targets in ICU-admitted patients, and seven were assessed in patients with acute myocardial infarction and stroke. For ICU patients, the evidence was conflicting, with some studies showing the efficacy of conservative oxygen therapy while others reported no difference. Overall, nine studies concluded that lower oxygen targets are favorable. However, most studies (n=4) in stroke and myocardial infarction patients showed no difference in lower or higher oxygenation targets whereas only two supported lower oxygenation targets. Available evidence suggests that lower oxygenation targets result in either improved or equivalent clinical outcomes compared with higher oxygenation targets.

## Introduction and background

The administration of supplemental oxygen is a crucial lifesaving strategy in emergency situation [1-3]. In the management of critically ill patients, achieving optimal oxygenation targets is crucial to ensure adequate tissue oxygen delivery while minimizing the risk of potential harm associated with both hypoxia and hyperoxia. During acute pathological conditions, such as cardiac arrest, myocardial ischemia, traumatic brain injury, and stroke, oxygen is liberally administered in a prehospital setting to mitigate the risk of tissue hypoxia [4,5]. Patients who survive the acute phase are usually admitted to the Intensive Care Unit (ICU) and receive mechanical ventilation [6]. In this setting, the fraction of inspired oxygen (FiO2) frequently exceeds the ambient air concentrations to mitigate tissue hypoxia [7]. This leads to supranormal arterial levels of partial pressure of oxygen (PaO2) or hyperoxia within the first 24 h of admission [8]. Although the detrimental consequences of hypoxia are widely recognized and are actively mitigated, hyperoxia is usually overlooked [9]. Furthermore, most healthcare providers view excessive supplementary oxygen administration as a harmless and potentially efficacious therapeutic intervention independent of the manifestation of hypoxemia [10]. This is partly due to the widespread use of supplemental oxygen therapy and the common perception that elevated PaO2 is a protective buffer against hypoxemia. Consequently, many ICU patients are at risk of oxygen over administration. The proclivity exhibited toward hyperoxia can be detrimental, as several clinical studies have shown adverse consequences [11]. Multiple mechanisms have been proposed to explain the potential harm associated with hyperoxia. Reactive oxygen species (ROS) generation, which can cause oxidative stress and tissue damage, is one such mechanism [12]. Excessive oxygen levels may also lead to vasoconstriction and impaired microcirculation, limiting tissue perfusion and contributing to organ dysfunction. Furthermore, hyperoxia has been implicated in mitochondrial dysfunction, inflammation, and immune dysregulation, which can ultimately exacerbate organ injury and impair patient outcomes [13].

A randomized controlled trial (RCT) indicated heightened early cardiac injury and increased myocardial infarct size in patients with ST-elevation myocardial infarction supplemented with oxygen who did not have hypoxia [14]. Moreover, hyperoxia has demonstrated both temporal and quantitative dependency in animal studies, which may result in adverse vascular permeability and proinflammatory pulmonary responses manifested as elevated levels of cytokines and chemokines in the pulmonary microenvironment [15,16]. Currently, the guidelines and optimal targets for oxygen supplementation are contradictory owing to the lack of definitive data and significant heterogeneity in published trial results [17,18]. Although an increasing number of RCTs have recently focused on demonstrating the efficacy of oxygenation targets, most studies have failed to provide substantial results for optimizing oxygenation targets [19,20]. A recent systematic review demonstrated a lack of consensus on the benefits and risks associated with low versus high oxygenation targets in patients admitted to the ICU [21]. Previously, numerous systematic reviews and meta-analyses that aimed to evaluate the same outcome measures had been published. However, these studies were subject to limitations primarily due to the incorporation of outdated literature [10, 19, 22]. As new data are continuously generated, it is imperative to maintain an updated body of evidence to provide clinicians and investigators with current guidance regarding crucial aspects of care. Therefore, this review aimed to summarize recent empirical data on optimal oxygenation targets and their impact on patient outcomes.

## Review

Materials and methods

The protocols for this review were devised in adherence to the guidelines prescribed by the Preferred Reporting Items for Systematic Reviews and Meta-Analyses (PRISMA) [23].

Search Strategy and Data Sources

For this review, comprehensive research was conducted using several databases to identify relevant studies that compared lower and higher oxygenation targets in critically ill patients. We performed separate searches in PubMed (seeking studies published from 2010 to 2023), MEDLINE (2010 to 2023), and Scopus (2010 to 2023). The research was conducted using a combination of different keywords, including “oxygenation targets,” or “oxygenation therapy,” or “high versus low oxygenation targets,” and “critically ill patients,” or “hypoxia patients.” Appendix 1 describes details of keywords used for the search. Related terms, alternatives, and plurals, such as hyperoxemia, oxygen supply, and fatality, were also considered. Furthermore, we searched Google Scholar and the reference section of the selected studies to increase the body of evidence in the current review. Studies that fulfilled the following criteria were included: (1) observed the efficacy of lower or higher oxygenation targets, (2) focused on the clinical outcomes of the intervention, and (3) published in English. Studies involving patients with chronic respiratory diseases, mental illnesses, extracorporeal life support, and hyperbaric oxygen therapy were excluded. As we focused on identifying the efficacy of high versus low interventions, we excluded studies that included surveys. Moreover, studies focusing on pediatric populations were excluded.

Data Collection Process

All retrieved articles from the database and Google Scholar searches were transferred to the reference manager (EndNote 20, Thomson Reuters) after excluding duplicate and non-English titles. Subsequently, the Endnote file was transferred to Rayyan, a web-based software package, to expedite the initial screening of the search results [24]. The data extraction process was performed by two blinded reviewers to ensure rigorous and unbiased evaluation of the included studies. The process was categorized into the following three stages: (1) the selection of studies based on titles and abstracts eligible for inclusion in the review, (2) a thorough analysis of eligible articles, keeping in mind the aim of the review, and (3) further searches were refined based on the exclusion and inclusion criteria, and data were obtained in the form of notes regarding the interventions used in the studies, number of participants, and methods used. After independent data extraction, the reviewers compared their results. Any discrepancies or disagreements were resolved through discussion and consensus. When consensus was not reached, a third reviewer was involved to make a final decision.

Flow Diagram

**Figure 1:**
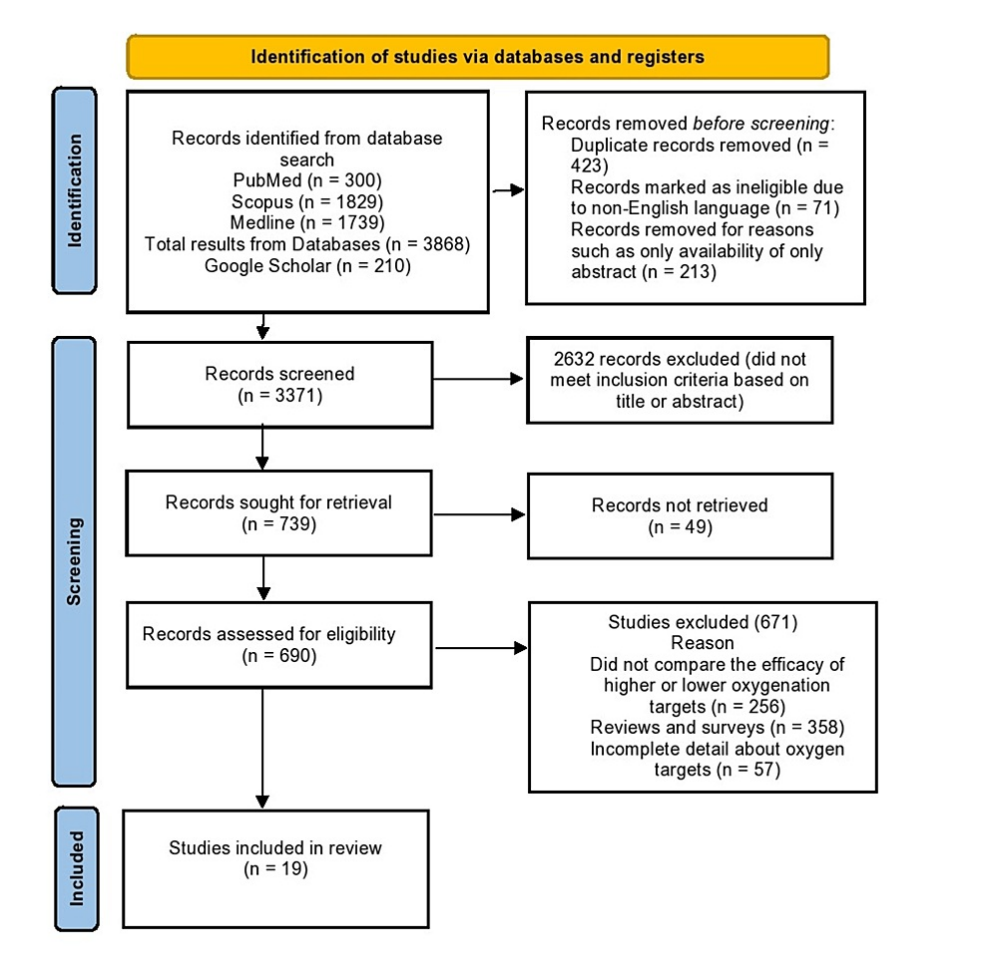
Preferred Reporting Items for Systematic Reviews and Meta-Analyses (PRISMA) Flow Diagram of the Systemic Review

The study design adhered to the PRISMA flow diagram and protocol [25], outlining the systematic approach of identifying relevant articles to select those that met the eligibility criteria for further analysis (Figure 1).

Results

Included Studies

The literature search provided 4,078 potentially relevant articles from the MEDLINE (n=1,739), PubMed (n=300), Scopus (n=1,829), and Google Scholar (n=210) databases. After excluding duplicate studies and non-English publications, only 3,371 records were analyzed. Based on abstracts and keywords, 2,632 publications were removed from the scope of this review. A thorough assessment of the residual corpus of literature was performed to identify the 19 most relevant studies for inclusion in the scope of this review.

Study Characteristics

Of the 19 studies reviewed, 12 investigated the efficacy of conservative oxygen strategies against established standards of oxygenation practices in a heterogeneous patient population in ICU settings. The remaining seven studies contrasted the outcomes of low and high oxygenation therapies in patients with acute myocardial infarction and stroke.

Lower Versus Higher Oxygenation Targets in ICU Patients

In the current review, conflicting evidence in the literature search regarding the efficacy of oxygenation targets, with some studies showing the efficacy of conservative oxygen therapy [26-32] whereas others reported no difference [33-35] (Table 1). A total of eight RCTs evaluated the oxygenation targets in ICU patients. The most common outcomes measured in the studies included ventilator-free days, mortality rates, and improvement in oxygen saturation after intervention. 

**Table 1:** Summary of recent studies that compared low versus high oxygenation targets in ICU patients FiO2, fraction of inspired oxygen; ICU, Intensive Care Unit; eICU-CRD, eICU Collaborative Research Database; MIMIC, Medical Information Mart for Intensive Care III database; IQR, interquartile range; LOS, length of stay; mRS, modified Rankin Scale; PaO2, supranormal arterial oxygen; RCT, randomized controlled trial; SaO2, saturation of arterial oxygen; SpO2, saturation of peripheral oxygen.

Study	Design	Sample Size	Reason for admission	Intervention	Main Findings	Conclusion
Mackle et al. [33]	RCT	1,000	Acute brain disease, surgery	In conservative intervention, the limit for SpO2, which was 97% FiO2, decreased to 0.21.	No significant difference in ventilator-free days. A difference of >28 h was reported duration of ICU stay in the conservative group and control group. Conversely, the conservative-oxygen cohort exhibited a diminished duration of time with SpO2 >96% (median time: 27 h [IQR: 11.0–63.5]) compared to 49 h [IQR: 22–112] in the usual-oxygen cohort). At 180 d, mortality rates were insignificant in both groups.	No difference was found between the conservative and normal groups.
Panwar et al. [26]	RCT	103	Trauma, Surgery, and medical	SpO2 levels 88–92% (conservative); SpO2 levels ≥96% (liberal).	SpO2, SaO2, PaO2, and FiO2 significantly differed between the conservative and liberal oxygenation groups (P<0.0001). No significance difference was found in mortality or organ dysfunction. The conservative arm had a higher percentage of time spent with SpO2 <88%.	Conservative oxygenation therapy is feasible compared to liberal.
Young et al. [27]	Post hoc analysis	251	Sepsis	In conservative intervention, the limit for SpO2, which was 97% FiO2, decreased to 0.21.	Patients with sepsis in conservative oxygenation therapy exhibited reduced time in ICU with SpO2 ≥97%. No significant difference was reported in 90 d mortality rates.	Conservative therapy is a better option in patients with sepsis.
Helmerhorst et al. [28]	Single-center pilot prospective before-and-after trial	15,045	N/A	Conservative oxygenation (PaO2: 55–86 mmHg).	PaO2 levels elevated from 47% at baseline to 63% and 68% during phases 1 and 2, respectively (P<0.0001). No significant differences in ICU fatality or ICU-free days were noted.	Conservative oxygenation targets were feasible.
van den Boom et al. [36]	Replicate retrospective analyses	26,723 in eICU-CRD and 8,564 in MIMIC	Atrial fibrillation, sepsis, stroke	–	A significant inverse relationship was observed between the time spent within the optimal SpO2 range and hospital mortality, with an odds ratio of 0.42 for eICU-CRD and 0.53 for MIMIC.	The most suitable range of SpO2 was 94–98%.
Suzuki et al. [29]	Pilot before-and-after trial	105	Cardiovascular, sepsis, respiratory	Conservative = SpO2 of 90–92%.	Time-weighted average SpO2 and PaO2 levels were significant between conservative and conventional oxygen therapy. The median SpO2 was 95.5% and 98.4% during conservative oxygen and conventional therapy, respectively (P<0.001). No significant differences were observed in the PaO2/FiO2 ratio or any other biochemical or clinical outcomes between the two therapy periods.	Conservative oxygen was more suited in terms of clinical outcomes.
Gelissen et al. [34]	RCT	400	Systemic infection, stroke, cardiac arrest, pneumonia	PaO2 = 8–12 kPa (low-normal); PaO2 = 14–18 kPa (high-normal).	No significant difference was reported in both groups regarding the median duration of mechanical or in-hospital mortality. Mild hypoxemia occurrences were more frequent in the low-normal group.	No difference was found in low or high oxygenation targets.
Girardis et al. [30]	RCT	434	Surgical, medical	Conservative therapy = SpO2 94–98%; Conventional therapy = SpO2 97–100%.	The conventional group exhibited higher median PaO2 than the conservative group (P<0.001). The conservative arm showed decreased mortality (11.6%), whereas conventional therapy showed increased mortality (20.2%).	Conservative protocol was better in terms of ICU mortality.
Azoulay et al. [37]	RCT	776	Acute hypoxemic respiratory failure	PaO2 <60 mmHg.	Patients who received high-flow oxygen therapy had a higher PaO2:FiO2 ratio and a lower respiratory rate after 6 h. No significant differences were observed regarding LOS, infections, and dyspnea.	No difference was observed regarding mortality outcomes.
Schjørring et al. [35]	RCT	2,928	Pneumonia, Cardiac arrest, Myocardial infarction, Traumatic brain injury	Lower oxygenation target = 60 mmHg; Higher oxygenation target = 90 mmHg.	At 90 d observation, the mortality rate was 42.9% vs. 42.4% in the low and high oxygenation target groups, respectively. No significant difference was observed between the groups regarding survival without life support or posthospital discharge survival rates.	No difference was found between low and high oxygenation targets.
Asfar et al. [31]	RCT	442	Sepsis	FiO2 at 1.0 (hyperoxia); SpO2 = 88–95% (normoxia)	At 28 d follow-up, 43% of patients in the hyperoxia group had died, while 35% of patients in the normoxia group had died. Adverse events were significantly different in both groups, with almost double the incidence observed in the hyperoxia group compared to the normoxia group.	Arterial hyperoxia increases the risk of mortality.
Taher et al. [32]	RCT	68	Traumatic brain injury	Experimental = 80% oxygen via mechanical ventilator; Control = 50% oxygen via mechanical ventilator.	The median duration of ICU stay was less in the experimental group (P=0.280). After 6 mo. of injury, the moderate outcome score was 16 and 9 in the control and experimental groups, respectively; mRS at discharge was 2.6 and 2.3 in the control and experimental groups, respectively (P=0.320).	Experimental oxygen therapy was better suited for critically ill patients.

Stroke and Myocardial Infarction

In the current review, four studies were performed on stroke patients [38-41] whereas three were reported on myocardial infarction patients [42-44] (Table 2).

**Table 2:** Summary of recent studies that compared low versus high oxygenation targets in stroke and myocardial infarction mRS, modified Rankin Scale; NBO, normobaric oxygen; RCT, randomized controlled trial.

Study	Design	Sample Size	Etiology	Intervention	Main Findings
Hofmann et al. [42]	RCT	6,629	Myocardial infarction	6 L/min (6–12 h) open-face mask or ambient air.	The median oxygen saturation was 99% in oxygen therapy compared to 97% in ambient air. The death outcome in both groups was insignificant, with 5.0% observed in the oxygenated group and 5.1% in ambient air patients.
Khoshnood et al. [43]	RCT	100	Myocardial infarction	Supplemental oxygen (10 L/min) or room air.	No difference was observed in infarct size.
Ali et al. [38]	RCT	289	Stroke	Treatment group: oxygenation at 2–3 L/min for 72 h; Control: room temperature.	At the end of 6 mo., the mortality rate was comparable between the two groups, with 22 (15%) and 20 (14%) patients dying in the oxygen and control groups, respectively.
Mazdeh et al. [39]	RCT	52	Stroke	Oxygen saturation 50%.	No significant difference was found in the constitutions of ischemic-hemorrhagic strokes between the two groups (P=0.200). No difference was observed in mRS (P=0.800).
Ranchord et al. [44]	RCT	136	Myocardial infarction	Oxygen saturation 93–96%.	The mortality rate doubled in the oxygen saturation group compared to the high concentration group. No significant difference in troponin T levels was observed between high-concentration oxygen and titrated oxygen.
Roffe et al. [40]	RCT	8,003	Acute stroke	Oxygenation via nasal tube (3 L/min). Oxygen saturation ≤93%. Rate = 2 L/min when oxygen saturation ≥93%.	Improved outcomes were observed in the higher oxygenation group.
Shi et al. [41]	Animal model	128	Stroke	100% oxygen (NBO) or normoxia 21% oxygen.	NBO showed a reduction in blood occluding levels with improved neurological outcomes.

Discussion

**Figure 2:**
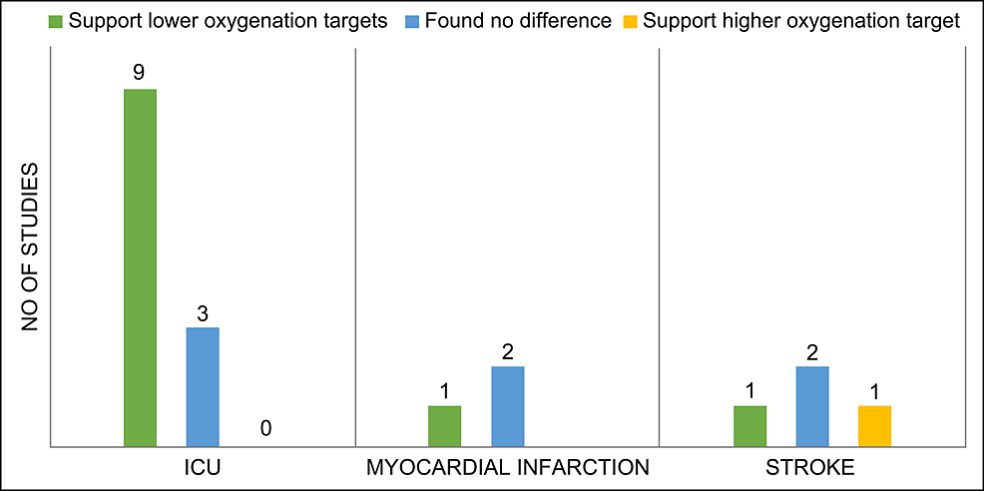
Summary of oxygenation-related conclusions in ICU, stroke, and myocardial infarction studies. ICU, Intensive Care Unit.

This review aimed to synthesize recent evidence in the field of oxygenation therapy for acute hypoxic conditions in critically ill patients. The review focused on synthesizing quantitative data and did not perform qualitative data analysis because this was beyond the scope of the study. The results revealed conflicting evidence regarding oxygenation targets in the literature search, which may be attributed to the diverse methodologies used in the clinical trials [36,40,45] The definition of normoxia for critically ill patients showed significant heterogeneity, with most studies defining it as a saturation of peripheral oxygen (SpO2) of 88-96% or a PaO2 of 60-150 mmHg. Among the 20 studies included in this review, hyperoxia or conventional treatment was defined as a PaO2 ranging from ≥120-300 mmHg, with occasional use of SpO2 ranging from ≥95-99%. A replicate retrospective analysis included in this review showed that the optimal range of SpO2 was 94-98% [36]. Most of the included studies in this review favored lower oxygenation targets and showed decreased adverse outcomes (Figure 2).

There has been a growing emphasis on conservative oxygenation interventions for ICU patients to reduce the potential hazards of hyperoxemia, as demonstrated in various studies [26-28,33]. Two RCTs by Panwar et al. [26] and Girardis et al. [30] propagated that the conservation oxygenation protocol demonstrated better ICU-related clinical outcomes. Panwar et al. showed that SpO2, PaO2, and FiO2 were significantly different between lower and higher oxygenation targets (P<0.0001). However, no significant differences were observed in the ICU length of stay or 90-day mortality outcomes between the groups. Similarly, Girardis et al. revealed a significant difference in the daily time-weighted average PaO2 levels; however, there was a lower incidence of mortality among patients receiving conservative oxygen therapy (11.6%) than among those receiving conventional oxygen therapy (20.2%). Furthermore, they reported a lower incidence of shock episodes and bloodstream infections. These findings are supported by several other studies conducted in ICUs. A prospective pilot study by Helmerhorst et al. [28] concluded that conservative oxygenation targets are feasible. They showed that episodes of hyperoxia declined significantly (P<0.0001), whereas that of hypoxic remained largely unchanged (P=0.060). Previously, a meta-analysis of randomized trials encompassing adult individuals with acute illnesses revealed that the unrestricted utilization of oxygen, without adhering to predetermined thresholds for arterial oxygen saturation, was found to be correlated with an elevated mortality rate compared to more controlled approaches [10].

A pilot study by Suzuki et al. [29] showed a significant difference in the median time-weighted average SpO2 and PaO2 levels between the lower and higher oxygenation targets. The median SpO2 was 95.5% during conservative oxygen therapy, compared to 98.4% during conventional therapy (P<0.001). However, no differences in the PaO2/FiO2 ratio or any other biochemical or clinical outcome were observed in either group [29]. Similarly, Asfar et al. [31] showed that the mortality risk increased with arterial hyperoxia. These findings have been contradicted by three studies included in this review [33-35]. The strongest evidence in this regard was provided by an RCT by Schjørring et al. [35], who found no difference in the clinical outcomes between the two interventions. They found no significant difference between the mortality rates in the lower oxygenation (42.9%) and higher oxygenation groups (42.4%). Furthermore, the study found no difference in the percentage of days alive without life support or after hospital discharge between the two groups [35]. These findings are further supported by the ICU randomized trial comparing two approaches to oxygen therapy (ICU-ROX) [33], which found no significant difference between the conservative and usual oxygen groups regarding ventilator-free days. Similarly, at the 180-day mark, the mortality rates were also insignificant, at 35.7% and 34.5% in the conservative- and usual-oxygen cohorts, respectively [33]. Although Gelissen et al. [34] found inconclusive evidence supporting higher or lower oxygenation therapy, they observed a higher rate of mild hypoxemia in the low-normal group than in the high-normal group. Previously, a correlation between low target oxygen saturation and an elevated incidence of episodic oxygen deprivation events has been reported [46].

The findings from this review show that the utilization of lower SpO2 targets is feasible and well tolerated, resulting in decreased pulmonary atelectasis [29], increased ventilator-free days, and reduced mortality [28]. Sepsis is a prevalent cause of critical care unit (CCU) admission and fatalities among critically ill patients [47]. This condition has been implicated in a substantial proportion of hospital fatalities, ranging from one-third to one-half [48]. In CCU settings, a significant subset of patients with sepsis require invasive mechanical ventilation; supplemental oxygen therapy is commonly employed among these patients. In this review, only one study focused on sepsis and favored the use of conservative oxygenation [27]. Among critically ill patients, tissue hypoxia is a frequent manifestation, which can exacerbate the likelihood of multiorgan system dysfunction. This phenomenon is characterized by decreased intracellular oxygen concentration, which reduces aerobic adenosine triphosphate generation [49]. Hypoxia is the most important manifestation of stroke [50,51] and myocardial infarction [52,53].

The present review provided indications of a potential advantage associated with the implementation of conservative oxygen therapy in individuals suspected to have hypoxic-ischemic encephalopathy. From a biological standpoint, it is plausible that conservative oxygen therapy can mitigate the occurrence of subsequent brain injury following resuscitation from cardiac arrest. Furthermore, observational data has indicated that exposing these patients to excessively high levels of oxygen (hyperoxemia) might have detrimental effects. In this review, we included six RCTs related to stroke and myocardial infarction in our investigation. In our literature search, we found two large RCTs by Hofmann et al. [42] and Roffe et al. [40]: one investigating myocardial infarction and the other examining patients with stroke. Hofmann et al. [42] compared the efficacy of oxygen therapy and ambient air in patients with myocardial infarction and reported the incidence of hypoxemia in 1.9% of patients in the oxygen group compared to 7.7% in the ambient air group. Similarly, Roffe et al. [40] reported better clinical outcomes in the oxygenated group than in the control group. The detrimental effects associated with hyperoxia have been demonstrated in animal studies to be contingent on both the duration and magnitude of exposure [54,55]. The potential for hyperoxia to exhibit dose-dependent adverse effects could not be conclusively determined based on the data analyzed in this review. Most studies examined either the initial PaO2/SpO2 level on admission or the maximum/minimum value during ICU or hospital stay, making it challenging to establish a clear relationship between the dose and oxygen toxicity. The limitations of this review include the lack of evidence of methodological studies that fulfilled the established inclusion criteria.

## Conclusions

This systematic review found a scarcity of high-quality studies that specifically examined the impact of high and low oxygenation targets in ICU patients. Despite this, available evidence suggests that lower oxygenation targets result in either improved or equivalent clinical outcomes compared with higher oxygenation targets. Clinicians should be aware that administering supplemental oxygen to non-hypoxemic patients results in only a negligible increase in systemic oxygen delivery; however, it may elicit significant adverse effects on various physiological processes, such as inflammation, oxidative stress, and pulmonary function. Additionally, the influence of normoxia on the incidence of hypoxic episodes has yet to be established, and the long-term consequences of restrictive oxygen therapy remain to be evaluated in extensive patient populations. Therefore, due to the absence of rigorous standards, further research is required to develop personalized oxygen targets for critically ill patients.

## Appendices

Appendix 1

Terms and strategy used for literature search.

**Table 3:** Terms and strategy used for literature search

#1 oxygenation targets	#10 OR/1-9
#2 oxygenation therapy	#11 critically ill patients
#3 higher oxygenation targets	#12 ICU patients
#4 lower oxygenation targets	#13 Severely ill patients
#5 high versus low oxygenation targets	#14 hypoxia patients
#6 lower oxygenation strategies	#15 Oxygen deprived patients
#7 Low-Normal Oxygenation Targets	#16 Patients in critical condition
#8 High normal oxygenation targets	#17 OR/11-17
#9 Low-Normal vs High-Normal Oxygenation Targets	#18 10 AND 17
